# Advances in music therapy for neurological rehabilitation

**DOI:** 10.3389/fneur.2026.1821915

**Published:** 2026-04-22

**Authors:** Miao Yu, Yuxuan Song, Haochong Song, Haoyang Duan, Guodong Zhang

**Affiliations:** 1 School of Music, Changchun University, Changchun, China; 2 School of Public Health, Tianjin Medical University, Tianjin, China; 3 College of Special Education, Beijing Union University, Beijing, China; 4 Department of Rehabilitation Medicine, First Hospital of Jilin University, Changchun, China

**Keywords:** auditory stimulation, mechanism of action, music therapy, neuroplasticity, neurorehabilitation

## Abstract

This review systematically examines the advances, mechanisms, and clinical applications of music therapy in neurorehabilitation. The article first delineates the conceptual origins of music therapy and the developmental trajectory of neurologic music therapy, introducing the major technical classifications including receptive methods, active methods, improvisational approaches, and vibroacoustic music therapy. The core section elaborates on the multidimensional mechanisms underlying music therapy in neurorehabilitation, encompassing neuroanatomical networks, neurotrans Melodic Intonation Therapy and endocrine systems, neuroplasticity, and psychobehavioral dimensions, elucidating the functional recovery pathways through auditory-motor integration, neural network reorganization, and neuroplasticity enhancement. Regarding clinical applications, the article comprehensively discusses the specific implementations and evidence-based efficacy of music therapy in the rehabilitation of stroke, Parkinson’s disease, and cerebral palsy, including improvements in motor, speech, and cognitive impairments. Finally, the article summarizes current challenges and outlines future directions, aiming to provide theoretical foundations and practical references for the standardized and precision application of music therapy in neurorehabilitation.

## 1 Introduction

Neurological disorders, including stroke, Parkinson’s disease, and cerebral palsy, have emerged as major global health concerns contributing to long-term disability and compromised quality of life. With the accelerating demographic transition toward aging populations and evolving disease spectra, the incidence and prevalence of these conditions continue to escalate, imposing substantial burdens on patients, families, and healthcare systems (1). Stroke, among the most prevalent neurological disorders, frequently results in multifaceted functional impairments encompassing motor, speech, cognitive, and emotional domains. Notably, over 70% of survivors experience varying degrees of limb paralysis, significantly compromising their activities of daily living and social participation (2). Parkinson’s disease is characterized by progressive bradykinesia, muscular rigidity, and postural instability; advanced stages commonly manifest gait abnormalities and increased fall risk, further restricting functional independence (3). Cerebral palsy, the most common motor disability in childhood, presents particularly prominent spastic manifestations, with persistent abnormalities in muscle tone and motor coordination substantially impeding developmental trajectories (4).

Confronted with these complex functional sequelae, neurorehabilitation aims to maximize functional restoration or compensation through systematic, individualized interventions to enhance patients’ quality of life. Although conventional rehabilitation approaches, including physical therapy, occupational therapy, and speech therapy, have demonstrated efficacy, they are frequently Melodic Intonation Therapy by protracted treatment courses, slow progression, and insufficient patient engagement and motivation (5). Furthermore, psychological disturbances, cognitive deficits, and social participation barriers often persist throughout the rehabilitation continuum, compounding the complexity and challenges of effective intervention (6). Consequently, the exploration of novel adjunctive therapeutic modalities capable of exerting multidimensional effects across physiological, psychological, and social domains has become a critical research priority in neurorehabilitation.

Music therapy, as an interdisciplinary, multimodal non-pharmacological intervention, has garnered increasing attention within the rehabilitation medicine community. Rooted in ancient human cultural and medical traditions, music therapy has evolved through the convergence of modern neuroscience, psychology, and rehabilitation medicine into a clinical application technology underpinned by systematic theoretical frameworks and empirical evidence. Through the scientific utilization of acoustic, rhythmic, and melodic elements, music therapy can directly modulate physiological states, emotional experiences, cognitive processes, and motor control, offering distinctive intervention pathways for neurological functional recovery (7).

In recent years, advances in neuroimaging, electrophysiology, and molecular biology have progressively elucidated the mechanisms underlying music therapy in neurorehabilitation (8). Research has demonstrated that music can regulate gait rhythm through auditory-motor entrainment, facilitate motor learning and Melodic Intonation Therapy via mirror neuron system activation, ameliorate mood and motivation through modulation of dopamine and endorphin release, and promote long-term functional recovery by enhancing neuroplasticity and brain network reorganization. These mechanisms provide theoretical foundations for the application of music therapy in post-stroke motor and speech rehabilitation, Parkinson’s disease gait training, cerebral palsy muscle tone modulation, and cognitive and emotional disorder interventions (9). This review aims to systematically examine the current status and advances of music therapy in neurorehabilitation, addressing its technical classifications, mechanistic underpinnings, and specific applications across various neurological disorders, thereby providing references for rehabilitation practitioners and informing directions for future high-quality clinical research and practice.

This review was conducted in accordance with the Preferred Reporting Items for Systematic Reviews and Meta-Analyses (PRISMA) guidelines. Two independent reviewers screened the literature published between January 2000 and January 2026. Only English-language studies involving human participants were included, with the aim of evaluating the effectiveness of virtual reality interventions for pusher syndrome after stroke. The search was performed in MEDLINE, Scopus, PEDro, and Web of Science using the strategy: (“Neurological Rehabilitation” OR “Neurorehabilitation” OR “Neural Rehabilitation”) AND (“Music Therapy”OR “Music Intervention”).

Inclusion criteria: ① Study design: randomized controlled trials, case series, or pilot studies comparing different interventions. ② Participants: patients diagnosed with neurological dysfunction, irrespective of age or sex. ③ Intervention and comparison: music therapy as the primary intervention, music therapy combined with other therapies, comparison with alternative techniques, or no comparator (case series).

Exclusion criteria: ① Full text not available. ② The experimental group received additional non-music therapy interventions on top of the control group’s treatment. ③ Duplicate publications.

## 2 Concept, origin, and development of music therapy

### 2.1 The concept of music therapy

Music therapy is internationally defined as “a systematic process of intervention wherein the therapist helps the client to achieve health goals through the use of musical experiences and the development of the therapeutic relationship” (10). This definition emphasizes its core characteristics of systematicity, professionalism, and relational orientation. The World Federation of Music Therapy further specifies that music therapy involves the evidence-based, planned use of music and musical elements by qualified music therapists to facilitate communication, expression, learning, and rehabilitation, thereby addressing individuals’ physical, emotional, psychological, social, and cognitive needs. It is not merely “treating illness through listening to music,” but rather a professional health intervention integrating receptive methods (e.g., listening, guided imagery), recreative approaches (e.g., singing, instrumental playing), and improvisational techniques.

### 2.2 Origins of neurologic music therapy

Neurologic Music Therapy represents a significant branch of music therapy at the intersection of neuroscience and rehabilitation medicine. Its emergence can be traced to the late 1980s and early 1990s, marked primarily by the research and clinical practice of Professor Michael Thaut and his team at Colorado State University, USA. The central tenet of Neurologic Music Therapy is neuro-mechanism-based musical intervention, emphasizing music as a “non-verbal neurostimulus” capable of directly engaging sensory, motor, cognitive, and emotional networks in the brain (11).

Through early electroencephalographic and behavioral studies, Thaut and colleagues systematically elucidated the “entrainment effect” of rhythmic auditory stimulation on the motor system—that is, external rhythmic cues can synchronize and optimize movement timing and coordination. Building upon this foundation, they developed techniques such as Rhythmic Auditory Stimulation and applied them to gait rehabilitation in patients with stroke and Parkinson’s disease, achieving remarkable efficacy (12). Concurrently, Marchina and colleagues, utilizing neuroimaging methodologies, demonstrated that singing and melodic intonation activate the right cerebral hemisphere, thereby providing novel theoretical pathways for aphasia rehabilitation and advancing the development of Melodic Intonation Therapy (13).

### 2.3 Development of neurologic music therapy

Since the initial establishment of its theoretical framework in the 1990s, Neurologic Music Therapy has undergone rapid and standardized development globally, manifested primarily in four dimensions.

#### 2.3.1 Deepening and expansion of theoretical frameworks

The original Neurologic Music Therapy model focused predominantly on auditory-motor pathways. With the widespread adoption of functional neuroimaging techniques (e.g., fMRI, PET) and electroencephalography, research has progressively revealed the effects of musical interventions on broader brain networks, including frontoparietal networks involved in cognitive control, limbic structures (e.g., amygdala, hippocampus) subserving emotional processing, and molecular mechanisms promoting neuroplasticity (e.g., brain-derived neurotrophic factor modulation). This has propelled the theoretical foundation of Neurologic Music Therapy from singular entrainment theory toward a comprehensive model encompassing neuroplasticity, network reorganization, neurotrans Melodic Intonation Therapy regulation, and psychophysiological integration (14).

#### 2.3.2 Systematization of technical standards and establishment of certification frameworks

To ensure scientific rigor and clinical consistency, the Academy of Neurologic Music Therapy has established standardized training and certification systems for Neurologic Music Therapy. A qualified Neurologic Music Therapy practitioner must master approximately 20 standardized therapeutic techniques, comprehend their underlying neuro-mechanisms, and demonstrate capability for targeted application based on comprehensive neurofunctional assessments. This has substantially enhanced the professional credibility of music therapy within multidisciplinary neurorehabilitation teams and established robust foundations for collaborative practice (15).

#### 2.3.3 Continuous accumulation of evidence-based research

Entering the 21st century, randomized controlled trials and meta-analyses examining Neurologic Music Therapy have increased substantially. High-level evidence has confirmed the efficacy of Neurologic Music Therapy in improving gait parameters (velocity, cadence, symmetry) among patients with stroke, Parkinson’s disease, and multiple sclerosis. Furthermore, Neurologic Music Therapy has demonstrated superior potential compared to conventional speech therapy in promoting speech fluency in individuals with non-fluent aphasia. Research domains have expanded from motor and speech functions to encompass cognitive impairment, unilateral neglect, emotional regulation, and pain management within neurorehabilitation contexts (16).

#### 2.3.4 Global dissemination and cross-cultural integration

Neurologic Music Therapy practice has expanded from North America to numerous countries and regions across Europe, South America, Asia, and Oceania. Throughout this dissemination, Neurologic Music Therapy techniques have undergone exploratory integration with local cultural traditions and medical practices (e.g., Five Elements Music in Traditional Chinese Medicine) to accommodate diverse patient populations and sociocultural contexts. International academic exchange and collaboration have become increasingly frequent, collectively advancing frontier exploration and clinical translation within this discipline (17).

## 3 Primary technical classifications of music therapy

### 3.1 Receptive method

Receptive methods, also termed receptive music therapy, represent one of the most extensively applied therapeutic approaches. The core mechanism involves guided listening to specifically selected music to facilitate the induction of relaxation, focused attention, or targeted emotional states to achieve therapeutic objectives. This approach is particularly applicable for attention training, emotional regulation, stress management, and cognitive stimulation. In clinical practice, therapists frequently integrate music-guided imagery, wherein patients are guided through imaginal associations against a musical backdrop to explore subconscious processes, address psychological conflicts, or stimulate creativity. For neurorehabilitation patients, listening to personally preferred, positively valenced music has demonstrated efficacy in improving mood, reducing anxiety and depression, enhancing motivation, and potentially optimizing physiological and psychological states for subsequent rehabilitation training through modulation of limbic system and autonomic nervous system functions. This method emphasizes the integration of music with the listener’s subjective experiential state, constituting a significant pathway for accessing internal psychological landscapes (18).

### 3.2 Active methods

Active methods, or participatory music therapy, emphasize patients’ active engagement and creative musical participation. Patients directly engage in musical activities through singing, instrumental playing, music composition, or rhythmic body movement. Within neurorehabilitation contexts, this approach is extensively applied in training motor, speech, and cognitive functions. For instance, patients may train upper limb coordination and fine motor control through drumming or keyboard playing; improve respiratory control, vocalization capacity, and speech fluency through singing; and enhance gait, balance, and trunk control through rhythm-synchronized body movement. Active methods not only target specific functional impairments directly but also substantially enhance patient motivation, self-efficacy, and training adherence through immediate feedback, experiences of mastery, and social interaction inherent in musical activities, embodying the therapeutic philosophy of “rehabilitation through music” (19).

### 3.3 Improvisational methods

Improvisational methods refer to spontaneous, real-time musical creation and interaction between patient and therapist using simple instruments or vocalization without predetermined structural constraints. The essence of this approach lies in the process rather than the product, emphasizing the expression of present-moment emotions, establishment of non-verbal communication, and exploration of interpersonal patterns through improvisational musical dialogue. In neurorehabilitation, improvisational methods hold distinctive value for ameliorating mood disturbances, facilitating social interaction, enhancing self-expression capabilities, and improving cognitive flexibility. For patients with expressive speech difficulties secondary to brain injury, improvisational playing provides an alternative channel for communication and emotional discharge. Therapists employ techniques such as synchronization, mirroring, or providing supportive harmonic frameworks to establish deep therapeutic alliances, promoting psychological and emotional integration within secure musical structures.

### 3.4 Vibroacoustic music therapy

Vibroacoustic music therapy constitutes a specialized form of music therapy integrating auditory and tactile-vibratory sensory experiences. It utilizes specially processed “vibroacoustic music” rich in low-frequency acoustic waves (typically 16–150 Hz), delivered through dedicated equipment such as vibroacoustic beds, cushions, or vests to Melodic Intonation Therapy physical vibrations directly to the body. Patients perceive musical rhythm and melodic vibrations through the entire body rather than auditory perception alone. This therapeutic modality is grounded in biophysical “resonance” principles, positing that specific frequency acoustic vibrations can generate harmonic resonance with human tissues and cells, thereby inducing muscle relaxation, spasm reduction, pain alleviation, improved circulation, and autonomic nervous system modulation. In neurorehabilitation, vibroacoustic music therapy has demonstrated significant efficacy in reducing elevated muscle tone, ameliorating spasticity, relieving chronic pain, and promoting deep relaxation in patients with cerebral palsy, post-stroke conditions, or spinal cord injury, providing an effective passive intervention modality for severely impaired patients unable to actively participate in musical activities (20).

## 4 Mechanisms of action underlying music therapy in neurorehabilitation

The therapeutic efficacy of music therapy in neurorehabilitation derives from its multi-target, multi-pathway mechanisms of action. Contemporary neuroimaging and neuroelectrophysiological research has elucidated that music can achieve comprehensive rehabilitation of motor, cognitive, speech, and emotional functions through activation of extensive brain networks, modulation of Melodic Intonation Therapy release, and promotion of neuroplastic changes.

### 4.1 Neuroanatomical and network mechanisms

#### 4.1.1 Hierarchical processing pathways of the auditory system

The auditory system serves as the primary gateway for musical information entering the brain, and its hierarchical processing structure provides critical support for the neural plasticity underlying music therapy. Acoustic signals, after being transmitted from the inner ear, pass sequentially through subcortical structures including the cochlear nucleus, superior olivary complex, lateral lemniscus, inferior colliculus, and medial geniculate body, ultimately projecting to the primary auditory cortex. Throughout this process, each level does not simply relay information but progressively extracts temporal, affective, and structural features of sound (21, 22). This hierarchical organization holds significant importance for neurorehabilitation: when a particular level is compromised due to pathological changes, musical information can still be processed compensatorily through parallel pathways, offering a potential neural alternative for the recovery of auditory function after stroke.

#### 4.1.2 Functional connectivity of auditory-motor systems

Extensive neural connections exist between the auditory and motor systems, constituting a direct pathway through which music therapy improves motor function. The auditory cortex forms functional connections with the premotor cortex, supplementary motor area, and cerebellum. Auditory information can also descend via the reticular formation to spinal motor neurons, forming an auditory-motor reflex loop. The theory of “rhythmic entrainment” provides the core mechanism for this process: external rhythmic musical stimuli can serve as a reference oscillator, coupling with internal motor rhythms to optimize the temporal structure of movement. Clinical studies have shown that rhythm-based auditory stimulation significantly improves gait stability in patients with Parkinson’s disease and post-stroke hemiplegia, with effects superior to visual or tactile stimulation. This superiority is closely associated with the auditory system’s higher temporal resolution and its more direct auditory-motor connections (12).

#### 4.1.3 Activation and remodeling of cerebral functional networks

Music processing does not rely on isolated brain regions but is achieved through the coordinated activity of large-scale functional networks. Functional magnetic resonance imaging and positron emission tomography studies consistently demonstrate that music engagement broadly activates the sensorimotor network, executive control network, default mode network, and the limbic system (23). The synergy among these networks not only supports music perception and execution but also provides the foundation for neural plasticity induced by musical training. For instance, early music listening after stroke can enhance functional connectivity between the default mode network and the executive control network, which is significantly correlated with improvements in patients’ cognitive function. Furthermore, when the left-hemisphere language network is damaged, homologous regions in the right hemisphere can participate in compensatory reorganization of language function through musical processing. This mechanism provides a direct theoretical basis for the application of Melodic Intonation Therapy in aphasia rehabilitation.

### 4.2 Neurotrans Melodic Intonation Therapy and endocrine mechanisms

#### 4.2.1 Activation of the dopaminergic reward system

Music possesses potent reward and motivational properties, with its neural substrate rooted in the activation of the mesolimbic dopaminergic system. Listening to pleasurable music induces striatal dopamine release, with dopaminergic release magnitude demonstrating positive correlation with the degree of musical pleasure experienced. This effect involves the reward pathway projecting from the ventral tegmental area (VTA) to the nucleus accumbens and prefrontal cortex.

In neurorehabilitation, dopaminergic system activation carries multiple implications. First, dopamine serves as a critical modulatory neurotrans Melodic Intonation Therapy for motor function; its release enhances motor cortical excitability and movement output fluency, which is particularly significant for ameliorating motor disorders in Parkinson’s disease and post-stroke conditions. Second, dopamine participates in motivation and reward anticipation; music-induced dopamine release can enhance patients’ intrinsic motivation for rehabilitation training and improve treatment adherence. Third, dopamine exerts modulatory effects on cognitive functions (e.g., working memory, cognitive flexibility), contributing to post-stroke cognitive impairment recovery (24).

#### 4.2.2 Cholinergic system and cognitive enhancement

Acetylcholine represents another neurotrans Melodic Intonation Therapy intimately associated with musical effects, playing a central role particularly in cognitive function modulation. Basal forebrain cholinergic neurons project extensively to the cerebral cortex and hippocampus, regulating attention, memory encoding, and learning processes. Research has demonstrated that musical stimulation can activate the basal forebrain, increasing acetylcholine release in the cortex and hippocampus, thereby enhancing information processing efficiency and synaptic plasticity.

This mechanism holds particular significance in the rehabilitation of post-stroke cognitive impairment. Wu et al. (in their review) indicated that music therapy may improve attention, memory, and executive function in stroke patients through cholinergic pathways. Furthermore, interactions between acetylcholine and *γ*-aminobutyric acid (GABA) may participate in music-mediated modulation of cortical excitability, providing an explanatory framework for the antiepileptic effects of music.

#### 4.2.3 Neuroendocrine modulation of stress responses

Neurorehabilitation patients frequently exhibit significant stress responses, characterized by hyperactivation of the hypothalamic–pituitary–adrenal (HPA) axis, elevated cortisol levels, and sympathetic nervous system overactivation. Chronic stress states not only compromise mood and sleep but also inhibit neuroplasticity and exacerbate neural injury. Music therapy modulates stress responses through multiple pathways: on one hand, music can reduce HPA axis activity, decrease cortisol secretion, and attenuate glucocorticoid neurotoxicity toward hippocampal neurons; on the other hand, music can enhance parasympathetic nervous system activity, reduce heart rate and blood pressure, and promote psychophysiological relaxation.

#### 4.2.4 Activation of endogenous analgesic systems

Pain and muscle spasticity constitute common challenges in neurorehabilitation; music therapy can exert ameliorative effects through activation of endogenous analgesic systems. Research has demonstrated that music listening can promote the release of endogenous opioid peptides, including endorphins and enkephalins. These neuropeptides bind to *μ*-opioid receptors, producing analgesic and euphoric effects. Additionally, music may influence pain gating mechanisms through modulation of serotonergic and noradrenergic systems.

In vibroacoustic music therapy, low-frequency vibrations (16–150 Hz) can directly stimulate mechanoreceptors and proprioceptors, modulating muscle tone and alleviating muscular spasms through spinobulbar-cortical pathways.

### 4.3 Neuroplasticity mechanisms

#### 4.3.1 Music training-induced structural plasticity

Neuroplasticity constitutes the core mechanism of neurorehabilitation, with music serving as an effective stimulus for inducing plastic changes. Longitudinal neuroimaging investigations have demonstrated that long-term musical training can induce significant cerebral structural alterations, including increased gray matter volume in the motor cortex, auditory cortex, corpus callosum, and cerebellum, as well as improved integrity of white matter fiber tracts such as the arcuate fasciculus and corticospinal tract (25).

These structural modifications reflect microscopic plasticity processes including synaptogenesis, dendritic spine remodeling, and myelination. The distinctive characteristic of musical training lies in its multimodal nature, simultaneously involving auditory perception, fine motor control, memory encoding, and emotional processing; this multi-system coordinated activation can generate more extensive and enduring plasticity effects.

#### 4.3.2 Promotion of post-stroke functional reorganization

In stroke rehabilitation, music therapy promotes functional reorganization through “use-dependent” plasticity principles. Specific mechanisms include: (i) facilitating neurological functional recovery in peri-infarct penumbra regions; (ii) enhancing contralateral hemispheric compensatory activation; and (iii) inducing functional reorganization in homologous regions. Music may also provide molecular substrates for neuroplasticity through modulation of neurotrophic factor expression (e.g., brain-derived neurotrophic factor [BDNF]) (26). Furthermore, the rhythmic framework of music can provide external temporal structure for motor learning, accelerating skill acquisition and consolidation.

#### 4.3.3 Involvement of the mirror neuron system

The mirror neuron system (MNS) represents an important mechanism for understanding musical action observation and execution. Distributed across the premotor cortex, inferior parietal lobule, and superior temporal sulcus, the MNS is activated during both observation of others’ actions and execution of similar actions. In musical activities, whether observing performance movements or personally engaging in playing, MNS activation occurs, forming an “observation-execution matching” mechanism.

### 4.4 Psychological and behavioral mechanisms

#### 4.4.1 Emotional regulation and motivation enhancement

The impact of music on emotion represents one of its most prominent psychological effects, involving extensive limbic system activation. The amygdala participates in rapid evaluation of musical emotion, the hippocampus is associated with music-related memory retrieval, and the cingulate cortex is responsible for emotional regulation and conflict monitoring. Music can elicit positive emotional experiences, counteract depression and anxiety, provide channels for emotional discharge and expression, enhance emotional regulatory capacity, and improve psychological resilience.

At the motivational level, the reward properties of music can enhance the attractiveness of rehabilitation training, reduce training-related fatigue, and prolong training duration, thereby generating cumulative rehabilitation benefits (27).

#### 4.4.2 Optimization of attention and arousal states

Music modulates cortical arousal levels through the reticular activating system, optimizing attentional states. Fast-tempo, high-arousal music can enhance alertness and attentional concentration, applicable for patients with attentional deficits or low arousal states; slow-tempo, low-arousal music promotes relaxation and attentional recovery, suitable for anxious or hyperaroused patients. This bidirectional regulatory effect renders music a flexible state-modulation tool.

#### 4.4.3 Enhancement of social interaction and self-efficacy

Group musical activities provide structured, supportive environments for social interaction. Collaborative music creation and performance can facilitate cooperative behavior, enhance group cohesion, and reduce social isolation. Concurrently, successful experiences in musical activities can enhance self-efficacy—the individual’s belief in their capability to accomplish specific tasks—an important psychological factor predicting rehabilitation outcomes. Progressive skill improvement achieved through music can establish positive feedback loops, sustaining long-term rehabilitation motivation.

## 5 Applications of music therapy in neurorehabilitation

As an evidence-based rehabilitation modality, music therapy has been extensively applied in the rehabilitation of various neurological disorders. Its multi-target, non-invasive characteristics render it an important adjunct to conventional rehabilitation approaches, demonstrating distinctive advantages in ameliorating motor, speech, cognitive, and emotional functions.

### 5.1 Stroke rehabilitation

Stroke constitutes the leading cause of adult disability in China, with approximately 70–80% of survivors experiencing residual functional impairments of varying severity (28). Research on music therapy applications in stroke rehabilitation is the most comprehensive, encompassing multiple dimensions including motor, speech, cognitive, and psychological domains.

#### 5.1.1 Motor function rehabilitation

Music-supported therapy represents an effective technique for improving post-stroke upper limb function. This approach integrates instrumental playing with functional task training, promoting fine motor control and hand-eye coordination through rhythmic performance on instruments such as piano, percussion, or electronic keyboards. One study recruited 14 patients with post-stroke hemiparesis; following 12 sessions of percussion-based music-supported therapy over 6 weeks, patients demonstrated improvements in hand movement dexterity, fluidity, and velocity (29). Additionally, musical gloves have been applied in post-stroke upper limb rehabilitation, wherein patients trigger differential sound effects through grasping or pinching gestures, coordinating with interactive gaming activities synchronized to music. Research findings indicate that musical gloves produce superior upper limb functional outcomes compared to isolated hand training in stroke patients during the recovery phase (30).

Rhythmic auditory stimulation currently represents the most frequently implemented music therapy modality for lower Melodic Intonation Therapy motor rehabilitation in stroke patients. When musical rhythm serves as an external cue to assist patients in modulating movement patterns, movement quality becomes more standardized, with motor function significantly exceeding that of patients not receiving rhythmic musical cues, suggesting that musical rhythm may ameliorate abnormal movement patterns to a certain extent. Wang et al. guided middle-aged and elderly patients to perform walking training synchronized to therapist-played musical rhythms for 4 weeks in addition to conventional rehabilitation, demonstrating improvements in step length, cadence, and maximum walking velocity, with significant reduction in step length asymmetry between paretic and non-paretic limbs (31). One study enrolled subacute stroke patients with mean age approximately 63 years and first diagnosis within 3 weeks of onset, investigating rehabilitation efficacy of rhythmic auditory stimulation combined with conventional physical therapy. Results demonstrated that combined RAS training produced significantly greater improvements in Functional Ambulation Category scores compared to conventional therapy alone (32). Zhang et al. through meta-analysis, found that music therapy combined with conventional rehabilitation training yielded superior therapeutic effects on post-stroke motor impairment and executive function compared to conventional rehabilitation alone (33).

#### 5.1.2 Speech function rehabilitation

Melodic Intonation Therapy represents a classical music-based intervention for non-fluent aphasia. This method utilizes patients’ preserved singing capabilities, gradually transitioning to spontaneous speech production through melodic and rhythmic scaffolding. Zhang et al. conducted an 8-week randomized controlled trial wherein post-stroke aphasic patients in the experimental group received Mandarin Melodic Intonation Therapy training. Results demonstrated that, compared to the control group, experimental group patients achieved significantly greater improvements in naming ability, auditory comprehension, and speech fluency (34). Van der Meulen et al. implemented the first randomized controlled trial of Melodic Intonation Therapy for chronic post-stroke aphasia, with results indicating that therapeutic effects were restricted to trained content, failing to generalize to untrained vocabulary expression or daily verbal communication. Compared with previous investigations, Melodic Intonation Therapy efficacy in chronic aphasia was inferior to early post-stroke intervention, suggesting that early Melodic Intonation Therapy application may yield superior rehabilitation outcomes (35).

#### 5.1.3 Cognitive function rehabilitation

Post-stroke cognitive impairment occurs in 50–75% of patients, substantially compromising rehabilitation efficacy. Music therapy can improve attention, memory, and executive function through multi-network activation mechanisms. Baylan et al. implemented an 8-week music therapy intervention in 56 stroke patients; results demonstrated that, compared to a control group listening to self-selected audiobooks, patients receiving mindfulness combined with music listening or music listening alone exhibited significantly greater cognitive improvements, with mindfulness combined with music listening closely associated with attentional focusing, attentional control, and emotional regulation (36). Särkämö et al. randomized 60 stroke patients into control, language, and music groups to investigate effects of music intervention on cognition and mood. Results indicated that, in addition to pharmacological and conventional rehabilitation treatments, daily supplementation with over 1 h of therapist-selected verbal or musical listening materials effectively improved verbal memory function and enhanced attention, with durable long-term therapeutic effects (37). Raglio et al. compared standard care rehabilitation combined with active music therapy versus standard care rehabilitation alone in 19 stroke patients with mean age 72 years; results demonstrated that the combined music therapy group achieved significantly superior improvements in anxiety and depression compared to the rehabilitation-only group (38).

### 5.2 Parkinson’s disease rehabilitation

Parkinson’s disease is characterized by bradykinesia, muscular rigidity, postural instability, and gait abnormalities. Music therapy has demonstrated significant efficacy in ameliorating motor function in patients with Parkinson’s disease (39, 40).

Thaut et al. selected 60 Parkinson’s disease patients aged 62–82 years, presenting rhythmic auditory stimulation through embedded rhythmic cues in folk or classical music during walking. Results demonstrated that patients receiving auditory stimulation achieved significant improvements in walking velocity, cadence, step length, ankle dorsiflexion angle, fall risk index, and fear of falling compared to the non-stimulation group (41). Benoit et al. utilized familiar folk songs as background music to guide elderly Parkinson’s disease patients in walking, without explicitly requiring synchronization of gait with musical beats. Findings indicated that musical rhythm effectively facilitated movement initiation, increased step length, enhanced walking velocity, and improved gait coordination, postural control, and balance function (42). Patients engaged in walking-while-singing training; results demonstrated that this approach significantly enhanced rehabilitation adherence, transforming walking training into enjoyable daily routines. Although cognitive improvements were not significant, patients’ anxiety and depression were substantially alleviated (43).

### 5.3 Cerebral palsy rehabilitation

Cerebral palsy represents the most prevalent motor disability in childhood. Vibroacoustic music (VA) constitutes a class of therapeutically processed music rich in low-frequency sinusoidal rhythmic waves (16–150 Hz), delivered through vibroacoustic equipment, musical stimulation, and individualized rehabilitation protocols operating synergistically. In recent years, music therapy applications in cerebral palsy rehabilitation have garnered increasing attention.

Musical stimulation can induce pleasurable experiences and alleviate muscular spasms in children with cerebral palsy. This therapeutic modality achieves mind–body integration through dual sensory pathways of auditory and somatosensory-vibrotactile perception. Kwak et al. implemented vibroacoustic music intervention in children with spastic cerebral palsy; results demonstrated effective reduction in muscle tone, alleviation of muscular spasticity, and improvement in limb motor function, establishing that music as rhythmic auditory stimulation can directly modulate motor neural pathways, producing significant amelioration of gait patterns and walking stability in the lower extremities of children with cerebral palsy (44).

### 5.4 Dementia rehabilitation

Music therapy has a long-standing history of application in dementia rehabilitation. Lyu et al. randomized 288 dementia patients over 65 years into two groups: one engaging in singing or listening to familiar, preferred music, and the other reading lyrics from the same songs, with an intervention period of 3 months. Neuropsychiatric Inventory (NPI) assessments demonstrated that singing or music listening significantly ameliorated neuropsychiatric symptoms in severe dementia, including delusions, hallucinations, agitation, anxiety, euphoria, and apathy, while effectively reducing caregiver burden; moreover, the music intervention group exhibited superior efficacy compared to the lyric-reading group, further confirming the clinical value of music therapy for dementia patients (45).

Cho randomized 52 dementia patients aged 67–99 years into singing, music listening, and television-watching groups for a 4-week intervention. Quality of Life-Alzheimer’s Disease (QOL-AD) assessments indicated that the singing group achieved the most significant quality-of-life improvements. This effect may be attributed to deep breathing exercises during singing increasing blood oxygen supply, alleviating muscular tension, and promoting psychophysiological relaxation; furthermore, compared with passive music listening or television watching, singing provided patients with greater opportunities for social interaction, contributing to the maintenance of social functioning (46).

### 5.5 Epilepsy rehabilitation

Music therapy as an adjunctive treatment for epilepsy has attracted increasing attention in recent years, though its efficacy demonstrates substantial individual variability. Lin et al. administered Mozart sonata auditory stimulation to 58 children with partial seizures, with concurrent continuous electroencephalographic monitoring. Results demonstrated that 81% of patients exhibited mean 33% reduction in interictal epileptiform discharges, with generalized discharge patients showing the most significant reduction; however, 20% of patients demonstrated mean 14% increase in interictal discharges. No significant correlations were identified between patients’ gender, intelligence quotient, number of antiepileptic medications, or subjective responses to music and electrophysiological changes (*p* > 0.05), and the discharge-reduction effect was independent of alertness levels or specific emotional responses (47).

Dastgheib et al. through meta-analysis, further confirmed that 84% of patients exhibited significant reduction in interictal epileptiform discharges following Mozart music listening. This investigation additionally revealed that therapeutic response was closely associated with patients’ intelligence quotient levels, discharge types (generalized or focal), and epilepsy etiology (idiopathic), with high intelligence quotient, generalized/focal discharges, and idiopathic epilepsy patients demonstrating more pronounced responses to music therapy (48) (Table 1).

**Table 1:** Table of included studies.

References	Author/year	Sample size (*N*)	Intervention protocol	Conclusion
(29)	Street AJ/2015	14	Musical instrument playing and techniques	Patients demonstrated improvements in hand movement dexterity, fluidity, and velocity
(31)	Wang Y/2020	60	Music therapy	Music therapy to patients with ischemic stroke can improve their gait, walking ability, lower limb motor function, balance ability and treatment satisfaction
(32)	Gonzalez-Hoelling S/2021	55	Music-based rhythmic auditory stimulation (RAS)	The walking ability of subacute stroke patients might be improved with music-based RAS combined with conventional physiotherapy
(34)	Zhang X/2021	40	Melodic intonation therapy (MIT)	MIT based on music therapy is effective and valuable for the recovery of speech function in patients with non-fluent aphasia
(35)	vander Meulen I/2014	27	Melodic intonation therapy (MIT)	Patients with subacute severe nonfluent aphasia, language production treatment with MIT was effective
(36)	Baylan S/2018	56	Music listening	Music listening was most strongly associated with increased activity, memory reminiscence, and improved mood
(37)	Särkämö T/2008	54	Music listening	Music listening during the early post-stroke stage can enhance cognitive recovery and prevent negative mood
(38)	Raglio A/2017	38	Music therapy	Music therapy assessment showed a significant improvement over time of non-verbal and sonorous-music relationships
(41)	Thaut MH/2019	60	Rhythmic auditory stimulation (RAS) training	RAS training significantly reduced the number of falls in Parkinson’s disease and modified key gait parameters, such as velocity and stride length
(42)	Benoit CE/2014	15	Music training program with rhythmic auditory cueing	Musical rhythm effectively facilitated movement initiation, increased step length, enhanced walking velocity, and improved gait coordination, postural control, and balance function
(43)	Burt J/2020	30	Music-contingent gait training	Patients’ anxiety and depression were substantially alleviated
(44)	Kwak EE/2007	25	Rhythmic auditory stimulation	RAS does influence gait performance of people with spastic cerebral palsy
(45)	Lyu J/2018	298	Music therapy	Music therapy is effective in enhancing cognitive function and mental wellbeing
(46)	Cho HK/2018	37	Music therapy	Music therapy may be an effective non-pharmacological intervention in improving quality of life
(47)	Lin LC/2010	58	Music therapy (Mozart K.448)	It is possible to reduce the number of epileptiform discharges in some patients

## 6 Summary

Music therapy, as a multidimensional, non-invasive neurorehabilitation intervention, has accumulated substantial evidence from international clinical practice and research. Its unique advantages lie in its ability to provide systematic and individualized rehabilitation support for motor, cognitive, speech, and emotional impairments caused by neurological disorders such as stroke, Parkinson’s disease, and cerebral palsy, through mechanisms including auditory-motor integration, neuroplasticity promotion, and emotional regulation. In particular, the mature application of specialized techniques such as rhythmic auditory stimulation, Melodic Intonation Therapy, and vibroacoustic therapy has significantly enhanced the precision and engagement of rehabilitation interventions, effectively improving patients’ functional outcomes and quality of life.

However, while existing studies provide positive evidence, certain limitations cannot be overlooked. First, most clinical trials have small sample sizes and limited statistical power, making it difficult to support broadly generalizable conclusions. Second, there is considerable heterogeneity across studies in terms of intervention duration, frequency, music type, and outcome measures, which limits comparability and meta-analytic synthesis. Third, many studies lack long-term follow-up data, leaving the durability and long-term benefits of the interventions unclear. Finally, as most studies are conducted in highly controlled research settings, their generalizability to real-world clinical practice remains to be validated.

Of particular note is the generally insufficient description of the musical stimuli themselves in the existing literature. Most studies fail to clearly report specific parameters of the music used, such as rhythm, melodic structure, harmony, timbre, intensity, or musical genre, nor do they provide standardized definitions of the stimulus materials. This methodological limitation severely constrains the reproducibility of research and the comparability of interventions across studies, thereby undermining the validity of meta-analytic syntheses. Future research should prioritize precise characterization of musical stimuli at the methodological level and promote the development of uniform reporting standards, thereby providing a more robust foundation for the standardization and optimization of intervention protocols.

Future research should address these limitations by advancing the standardization and individualization of intervention protocols and conducting multicenter, large-sample, rigorously designed randomized controlled trials to systematically explore the optimal timing and dose–response relationships across different stages of disease. Additionally, further integration of music therapy with emerging technologies such as virtual reality and artificial intelligence should be pursued to develop intelligent, personalized rehabilitation models. Promoting the professional training of music therapists and integrating music therapy into clinical pathways will facilitate the broader, standardized application of this humanistic, low-cost rehabilitation approach within healthcare systems, ultimately benefiting a wider population of patients with neurological disorders and enhancing their quality of life and social participation.
